# Loretin, the New Antiseptic

**Published:** 1896-07

**Authors:** S. Eldred Gilbert

**Affiliations:** Philadelphia


### LORETIN, THE NEW ANTISEPTIC.

BY S. ELDRED GILBERT, D.D.S., PHILADELPHIA.

THE advantage of iodine preparations in antiseptic surgery have been firmly established, and that of iodoform has become the one most generally used, but with all of its good qualities there are several that are not so desirable. The disadvantages of its odor, toxic and irritant characters, greatly limit its use, and a new iodine preparation has long been desired and sought which shall be free from odor and absolutely non-poisonous. Loretin supplies this want. It has a complex constitution, being systematically called meta-iodo-ortho-oxyquinolin-ana-sulphonic acid and represented by the formula $C_9H_4IN.OH,SO_3H$. "Loretin is a bright yellow-colored crystalline powder not unlike iodoform in appearance. It is slightly soluble in water (about two parts in one thousand) and alcohol. It is practically insoluble in ether and in oils, but forms emulsions with oily liquids, and collodion, a soluble form of loretin, is also prepared. Being an acid it forms neutral salts with alkalies, which are easily soluble in water, forming solutions of an orange-red color. The calcium salt is insoluble in water, and can be easily precipitated on gauze impregnated with a solution of sodium salt by dipping into a solution of calcium chloride. Loretin gauze possesses the bright red color of this calcium salt." It may be employed as loretin powder, either alone or mixed in different proportions, with suitable materials, as calcined magnesia, French chalk, starch, etc.

Loretin colodion, in two- to ten-per-cent. emulsions.

Loretin pencils of cocoa butter, containing five to ten per cent.

Loretin ointment, five to ten per cent., with vaseline or lanolin.

Loretin solution, containing 0.1 to 0.2 per cent. of free acid or one to two per cent. or more of the soluble sodium.

Loretin gauze impregnated with precipitated calcium salt.

Its non-toxic property has been fully established by Professor Claus and Dr. Ammelburg by careful experiment, and borne out by clinical experience. Professor Schinzinger says he has employed loretin with great success in the treatment of boils, burns, lacerated wounds, poisoned cuts, and in gynæcological practice; also in many major surgical operations. In none of these was there a single instance of toxic effect.

In purulent discharges it quickly removes the offensive odor. Professor Schinzinger considers no praise too high for the antieczematous powers of loretin. He says that generally, when he has been called as consulting physician and prescribed loretin, the eczema disappeared where it had resisted all other treatment and which every proposed remedy had increased. Its favorable action, he says, was distinctly developed in one case recently under treatment. "Following a slight phlegmon on the elbow, treated with carbolic acid, an extremely painful eczema spread over the whole of the forearm and up to the shoulder. After different medicaments had been tried without success and the eczema had been considerably heightened by carbolic-zinc plaster, it completely disappeared in a short time under loretin treatment." In its uses Ammelburg says that he has never been able to find albumen, blood, sugar, or iodine in the urine. The absence of the latter is especially corroborated by Professor Albrecht. Before passing to the bacteriological proofs of this disinfectant, I wish to say that for the following tables I am indebted to Dr. B. Korff.

"To each twenty cubic centimetres of disinfectant three drops of a pure bacteria cultivation was added, or of pus, and allowed to remain with occasional shaking for twenty minutes; then from each mixture four test-tubes containing sterilized broth gelatin were inoculated, to the first tube 1 c.c. of the infected disinfectant, to the second and third ½ c.c. each, and to the fourth ¼ c.c. being added. They were well mixed and then poured out on cultivation plates."

TABLE I.: *Pure Cholera Cultivation.*—Meta-cresol, two per cent.: no growth. Carbolic acid (phenol. absol.), two per cent.: no growth. Lysol, two per cent.: no growth. Loretin, two per cent.: no growth. Loretin, two per mille: no growth. Iodoform, two per cent.: upon all four plates, scattered colonies. Control, numerous colonies.

TABLE II.: *Pure Staphylococcus Pyogenes Aureus Cultivation.*—Meta-cresol, two per cent.: no growth. Carbolic acid, two per cent.: no growth. Lysol, two per cent.: no growth. Loretin, two per cent.: no growth. Loretin, two per mille: no growth. Iodoform, two per cent.: isolated colonies. Control, numerous colonies.

TABLE III.: *Anthrax, Anthrax Broth.*—Meta-cresol, two per cent.: no growth. Carbolic acid, two per cent.: no growth. Lysol, two per cent.: no growth. Loretin, two per cent.: no growth. Loretin-sodium, one per cent.: no growth. Iodoform, two per cent.: isolated colonies. Control, numerous colonies. In this series a one-per-cent. aqueous solution of loretin-sodium was tried instead of a two-per-mille aqueous solution of loretin.

TABLE IV.: *Pure Bacterium Coli Cultivation.*—Meta-cresol, two per cent.: no growth. Carbolic acid, two per cent.: no growth. Lysol, two per cent.: no growth. Loretin, two per cent.: no growth. Loretin, two per mille, on all plates: numerous colonies; about half as many as with iodoform. Iodoform, two per cent., on all plates: numerous colonies. Control, innumerable colonies.

TABLE V.: *Pure Typhus Cultivation.*—Meta-cresol, two per cent.: no growth. Carbolic acid, two per cent.: no growth. Lysol, two per cent.: no growth. Loretin, two per cent.: no growth. Loretin-sodium, one per cent.: no growth. Iodoform, two per cent.: no growth. Control, numerous colonies.

TABLE VI.: *Pure Streptococcus Cultivation.*—Meta-cresol, two per cent.: no growth. Carbolic acid, two per cent.: no growth. Lysol, two per cent.: no growth. Loretin, two per cent.: no growth. Loretin-sodium, one per cent.: no growth. Iodoform, two per cent.: no growth. Control, numerous colonies.

TABLE VII.: *Pus from a very Infectious Phlegmon* (containing streptococci and staphylococci).—Carbolic acid, two per cent.: retarded growth of streptococus colonies. Loretin, two per cent.: no growth. Iodoform, two per cent.: retarded growth of streptococcus colonies. Control, very numerous colonies of streptococcus and staphylococcus.

By the foregoing we can readily see what a valuable preparation loretin is in surgery. It is just as valuable to the dentist in his line. For nearly a year I have been using it in the treatment of pulpless teeth and teeth having abscesses in all stages with and without fistulous openings, and where I have formerly used iodoform, etc., and am now ready and fully prepared, from clinical experience, to say that loretin is superior in my hands to any of the many antiseptics I have tried. One pleasant thing to me about it is that now the persistent odor of iodoform in my office is a thing of the past, and patients are no longer asking "what that horrid smelling stuff is?" no more nausea that would sometimes happen when iodoform was used.

The action of loretin is prompt in the treatment of putrescent teeth. I have had a great deal of pleasure in its use in the treatment of putrescent pulps, having used it in several ways, but most in combination with oil of cassia, as this is a pleasant vehicle for conveying the loretin into the tooth-canal.

It may be well to give the method employed in treating these teeth. After having prepared the tooth crown the root-canals should be well opened, going as near to the apex as possible; the medicament is then pumped into the root by means of a broach wound with cotton, then a string of cotton saturated with the loretin preparation is packed in the root and the patient dismissed for a week; upon the patient's return the cotton is removed (of course no saliva is allowed to enter the tooth), and if there is no odor other than that of the medicament, another string of cotton is placed in the root same as before, but this is covered with Gilbert's temporary stopping, closing it tightly; the patient is instructed to return in a week in case there is no inflammation, but should the tooth become sore to return immediately, when the temporary stopping is removed, as the tooth by this tenderness indicates that it is not yet in condition for filling; if the patient returns at the expiration of the week with no soreness in the tooth, it is ready for the temporary or trial filling, which is as follows: Form temporary stopping into cones to fill the canals; this is done by warming the stopping and rolling it between the thumb and finger; remove the dressing that is in the tooth, and with a cotton-covered broach pump in a little of the loretin mixture, following with chloro-stopping;[1] then insert the temporary stopping cones in the canals, filling the crown with white temporary stopping. This filling is generally allowed to remain for two weeks, at the end of this time we are sure that if there has been no trouble it is safe to fill permanently. In doing this the stopping in the roots is not disturbed but allowed to remain, as there is no better root-filling.

The above method is where a fistula has not been established; in case it has, the tooth is opened thoroughly, passing through the apex, the loretin mixture is pumped through the tooth until it appears on the gum through the fistula; this is followed by pumping the chloro-stopping through until it also appears on the gum. The temporary stopping cone is inserted in the roots, the tooth filled with white temporary stopping, and the patient dismissed for from three to six weeks or until the abscess has thoroughly healed, which is generally in about four weeks, unless there is carious bone or some cause other than simply the abscess. Of course, cases out of the common line are treated according to the cause. The above treatment has proved successful in many cases of long standing. Care should be used not to flood the crown of the tooth with too much loretin, as it has a tendency to darken it. I have had no trouble that has amounted to anything thus far, as I have tried to use care in this direction.

---

[1] Chloro-stopping is made by dissolving white temporary stopping in chloroform.

